# Golden ratio in venation patterns of dragonfly wings

**DOI:** 10.1038/s41598-023-34880-8

**Published:** 2023-05-15

**Authors:** Keene Lu, Samson Shen, Lisa M. Miller, Xiaojing Huang

**Affiliations:** 1 National Synchrotron Light Source II, Brookhaven National Laboratory, Upton, NY 11973 USA; 2 Ward Melville High School, Setauket-East Setauket, NY 11733 USA; 3 Computer Science Department, Northwestern University, Evanston, IL 60208 USA

**Keywords:** Biological physics, Applied mathematics

## Abstract

The vein pattern in insect wings allows this lightweight structure to carry multiple biological functions. Here, an investigation of the angular distribution of the vein struts in dragonfly wings revealed that the golden angle or golden ratio dominates the venation patterns. We find that the golden angle dominates the intervein angles in regions where thin veins and membranes demand strength reinforcement. A golden ratio partition method has thus been developed that explains a set of preferred intervein angles in distorted polygon-shaped venation cells throughout the venation pattern in dragonfly wings. These observations provide new evidence that the wing structure is spatially optimized, by the golden rule in nature, for supporting biomechanical functions of dragonfly wings.

## Introduction

Insect wings are constructed by strut-like veins that crisscross the wing surface and form unique geometric patterns. This lightweight architecture is stiff^1^, flexible^2^, fracture resistant^3^ and aerodynamically efficient^4^. Investigations of the shape^5^, morphology^6^ and micro-structure^7^ of insect wings suggest that those important functionalities are directly related to the venation arrangement.

Wing venation patterns are just one example of the great variety of recurring patterns in nature, all of which have developed over millennia via the biological processes of natural selection. Interest in mathematical modeling of these patterns dates to the early Greek philosophers such as Plato, Pythagoras, and Empedocles. Today, many of these patterns existing in nature can be classified as Turing patterns^8^, which can be explained by a widely recognized theoretical model of reaction-diffusion mechanisms with feedback loops that generate intricate patterns such as spots, stripes, and spirals during the morphogenesis of animals and plants^9–11^.

Another widely observed pattern in nature is the Fibonacci sequence, i.e., 0, 1, 1, 2, 3, 5, 8, 13 … (each subsequent number being the sum of the two preceding ones), which provides an optimization mechanism for the arrangement of, for example, leaves on stems and florets in flower heads^12,13^. In these cases, two rules govern the pattern formation: each element (1) bears the same spatial relationship to surrounding elements, and (2) inserts into the largest existing gap. When packing elements following these rules, the angular spacing between adjacent elements have been shown to be a division of two consecutive Fibonacci numbers of even order^14^. When the number of elements goes to infinity, this angular step size converges to $1/\phi ^2$, where $\phi$ is the golden ratio $(1+\sqrt{5})/2$. The corresponding angle $\sim 137.5^\circ$ is known as the golden angle *g*, which divides a full circle into 2 arcs in the golden ratio.

There are many examples in nature where these patterns effectively optimize a species’ functionality by evolving to the golden angle; for example, each leaf on an oak stem evenly receives sunshine and each petal on a sunflower is equally exposed to pollinators. And the golden angle extends well beyond plants and animals to the solar system, weather, music, and architecture^15^.

Characteristics of dragonfly (*Erythremis simplicicolis*) wings such as domain shape, size, and circularity have been widely studied^5,16^. How the vein pattern contributes to wing biomechanics has also been intensively investigated. The crossvein types and the cross/longitudinal vein links in dragonfly wings allow torsion and develop camber thus preventing transverse bending^17^. The vein microjoints provide local flexibility and reduce the load-induced stress concentration^18^. The three-dimensional structure of dragonfly wings such as corrugations and basal complexes increases the wing rigidity and improves the aerodynamics during flapping^19^. Theoretical modeling and empirical observations revealed the correlation between wing morphology and flight performance. For instance, narrow and broad wing bases are designed for low- and high-speed agilities, respectively^20^. Numerical models were developed to simulate the mechanical properties of the basal venation and basal complex to enable versatile flight capabilities^21^.

In this study, we focused on the angle as a basic geometric element and investigated the angular distribution of the vein pattern in the dragonfly wing. The results show that the golden angle or golden ratio indeed dominates in the angular distribution in the dragonfly venation patterns, likely contributing to their optimized biomechanical properties.

## Materials and method

**Figure 1:**
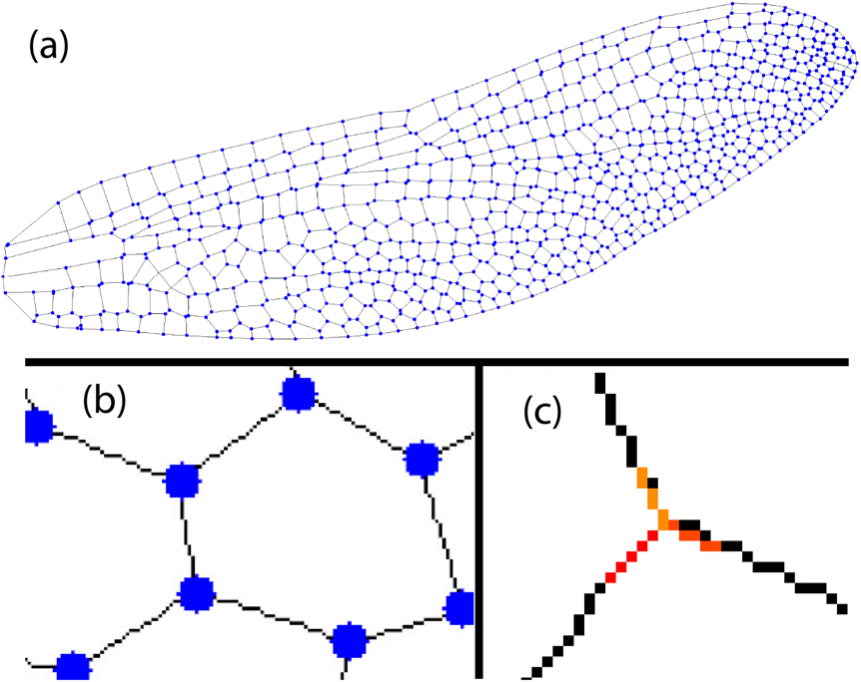
Intersection detection and angle calculation of a dragonfly wing. (**a**) The intersections are identified in a skeletonized dragonfly wing. (**b**) A zoomed-in view of the intersections is shown as blue dots. (**c**) The fitted lines surrounding the intersections shown as colored straight lines are used for calculating the angles between the veins.

Fifty high-resolution dragonfly wing images were used in this study, from the database provided in^16^. The images of the wings were individually extracted from the database, and were scaled up to twice their original size to grant a higher pixel count for the program to work with. They were then thresholded to black and white. The veins were represented as black lines of one-pixel width with a white background. Each skeletonized image was turned to the venations with a line of single-pixel-width, as shown in Fig. 1a. The intersections were detected by searching a $3\times 3$-pixel area around the vertex and locating connected pixels using a line-segmentation-based junction detection method^22,23^. For each vertex coordinate, a $7\times 7$ pixel area around the vertex is searched for black pixels, which represent the veins and form the intersection. To trace the trajectory of veins, reaching outwards from each vein intersection (as indicated as blue dots in Fig. 1b), a region defined by the minimum intersection distance ($15\times 15$ pixels) was surveyed, and the coordinates of every black pixel within the region were extracted. These coordinates were then grouped into individual veins defined as continuously connected pixels extending out from the vein intersections. Each vein was modeled as a straight line through a least-squares regression, as shown in Fig. 1c. The angle between adjacent veins was calculated at each intersection. It was estimated that the statistical uncertainty of the angle values determined from this least-squares regression method was about $\pm 1^{\circ }$, mostly due to the skeletonization process and the straight-line fits the vein segments.

## Results and discussion

**Figure 2:**
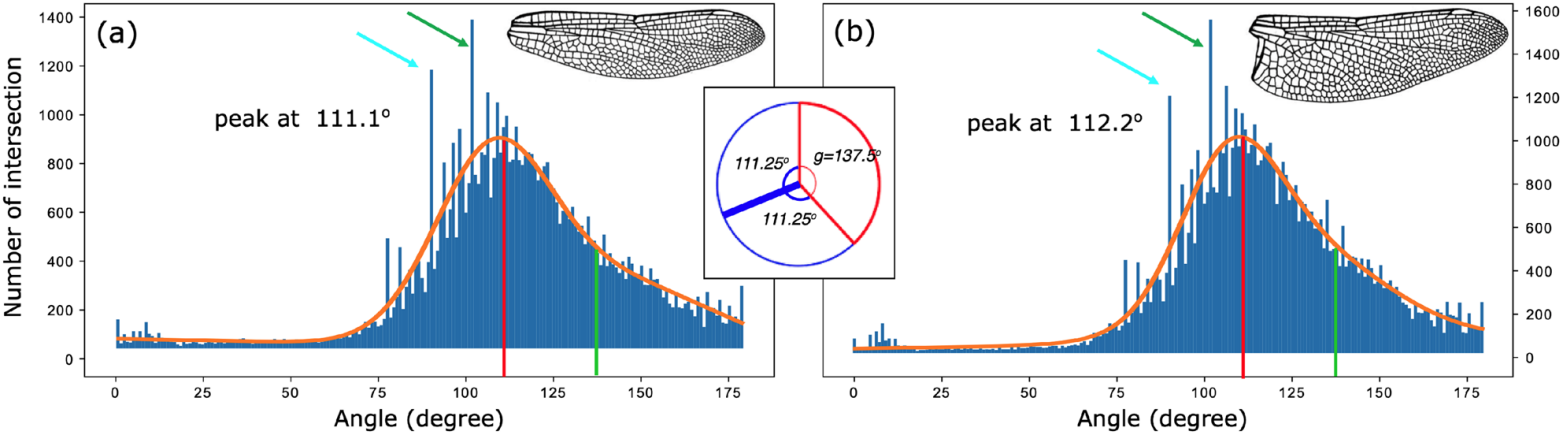
Angular distributions of dragonfly forewings (**a**) and hindwings (**b**). A summation of two Gaussian functions was used to fit the skewed histograms. The peaks of the fitted curves are located at 111.1$^\circ$ and 112.2$^\circ$, respectively. Considering that most vein intersections consisted of 3 veins, when the largest angle was the golden angle of 137.5$^\circ$, evenly splitting the remainder of the circle into two equal angles, as shown in the inset, the obtained angles would be 111.25$^\circ$, which agrees with the obtained histogram peak values. The cyan and green arrows indicate secondary peaks at 90$^\circ$ and 101$^\circ$, respectively.

The obtained angles for 50 dragonfly wings are plotted as histograms in Fig. 2. The distributions deviate from a Gaussian distribution, having an elevated tail on the high-angle side. This phenomenon implies that optimized biomechanical function does not necessarily require perfect symmetry. A summation of two Gaussian functions was used to fit the asymmetric angular distributions. For the forewings and hindwings, the fitted curves peak at $111.1^{\circ }$ and $112.2^{\circ }$, respectively. The relationship between the obtained peak and the golden angle *g* can be revealed by a closer observation on the venation pattern. From Fig. 1a, b one can see that most of the intersections consist of 3 veins and thus form 3 angles. A zoom-in view around a specific intersection, as shown in Fig. 1c suggests that, out of the 3 angles, one is slightly larger, while the other two are roughly equal. The statistics from the tested 50 wings show that the largest angle is the golden angle of $137.5^\circ$ and the two evenly split angles are $111.25^\circ$, as shown in the insert of Fig. 2. When the golden angle dominates in the venation pattern, the most abundant angle should be $111.25^\circ$, which agrees with the obtained histogram peak values. This relationship is precisely confirmed by the histograms, which show that the number of the peak angles ($111.1^{\circ }$ and $112.2^{\circ }$) almost exactly doubles ($2.2\pm 0.6$) the number of the golden angles ($137.5^{\circ }$). The dragonfly wing architecture is a three-dimensional structure, which has corrugations in the direction perpendicular to the wing surface^24,25^. The corrugation angle is less than 5$^{\circ }$ in most wing areas^16^, and the wing is almost flat near the tips^6^. The impact of corrugation is estimated to introduce an uncertainty of less than $1\%$ to the observed projection angles from the venation patterns. This effect may explain the slight difference between the obtained peaks at $111.1^{\circ }/112.2^{\circ }$ and the expected $111.25^{\circ }$.

**Figure 3:**
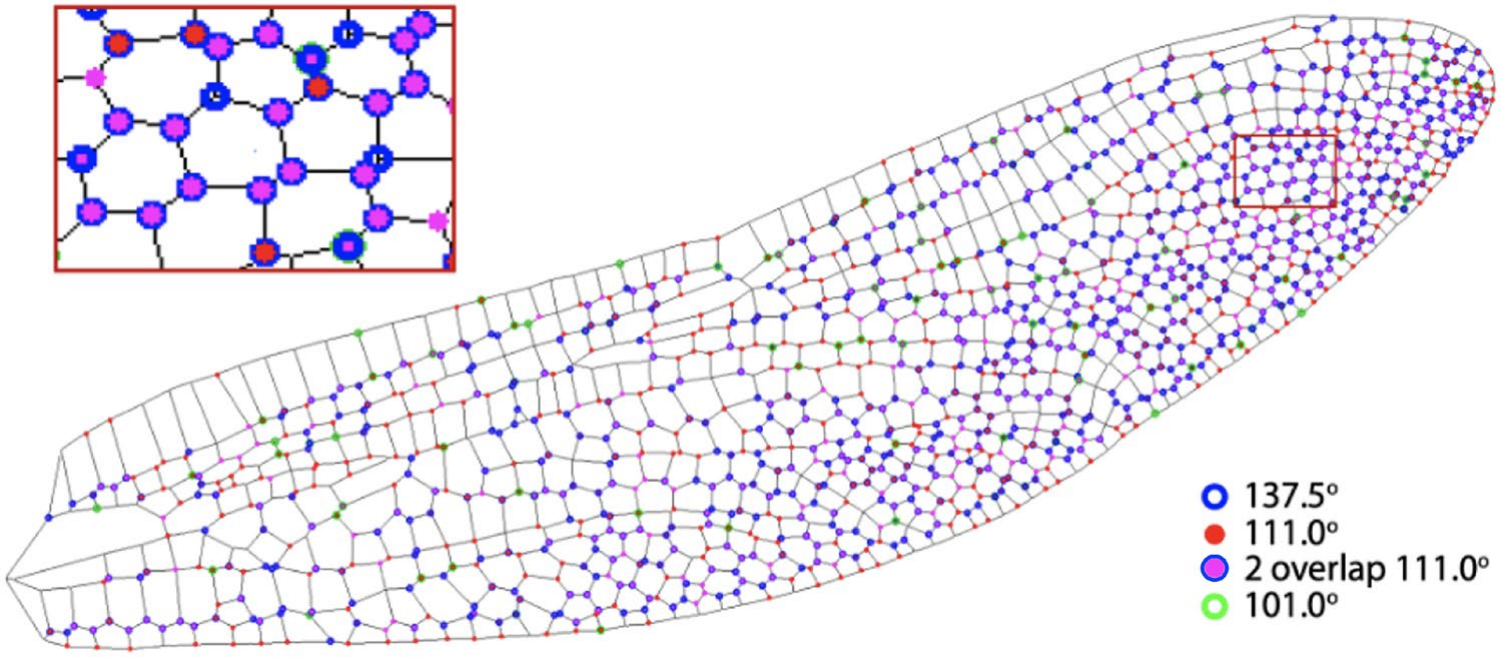
The locations of intersections for three specific angles on a dragonfly wing. Intersections that form $137.5^{\circ }(\pm 2.5^{\circ })$ and $111^{\circ }(\pm 2.5^{\circ })$ are labeled in blue and red, respectively. The intersections that have two $111^{\circ }(\pm 2.5^{\circ })$ angles are labeled in magenta. Most of the blue circles overlap with magenta dots, as shown in the inset. The angle splitting at these intersections exactly follows the golden ratio as illustrated in the inset of Fig. 2, and they are primarily observed near the trailing edge and the wing tip. Intersections forming $101^{\circ }(\pm 2.5^{\circ })$ are labeled in green, which identifies the interfacial areas connecting primary and secondary veins.

The locations of intersections forming $\sim 137.5^{\circ }$ and $\sim 111^{\circ }$ angles are indicated as blue and red circles in Fig. 3. The intersections that have two $111^\circ (\pm 10^\circ )$ angles are labeled in magenta. Most of the blue circles are overlapped with magenta dots, as shown in the inset. The angle splitting at these intersections exactly follows the golden ratio as illustrated in the inset of Fig. 2. These intersections are generally far away from primary veins and concentrate near the trailing edge and the wing tip. At these locations, both the vein and membrane are the thinnest^6^, so these are the regions where structural optimization is most needed for biomechanical enhancement.

In addition to the primary peaks in the histograms around 111.25$^{\circ }$, there are several sharp secondary peaks with the strongest two located at 101$^{\circ }$ and 90$^{\circ }$, as pointed by green and cyan arrows, respectively, in Fig. 2. The peak at 90$^{\circ }$ is mainly from the intersections in primary veins, which can be easily recognized in Fig. 1a, particularly near the wing boundaries. The primary veins are mainly constructed with rectangular patterns, which play an important role in guiding the direction of the spreading force^2^ and conducting hemolymph^26,27^. The peak at 101$^{\circ }$, on the other hand, is less obvious where they are mostly located, although Fig. 3 suggests that these (green circles) can be found at the interfacial region that connects the rectangle-dominated primary veins and the golden-angle-dominated secondary veins.

**Figure 4:**
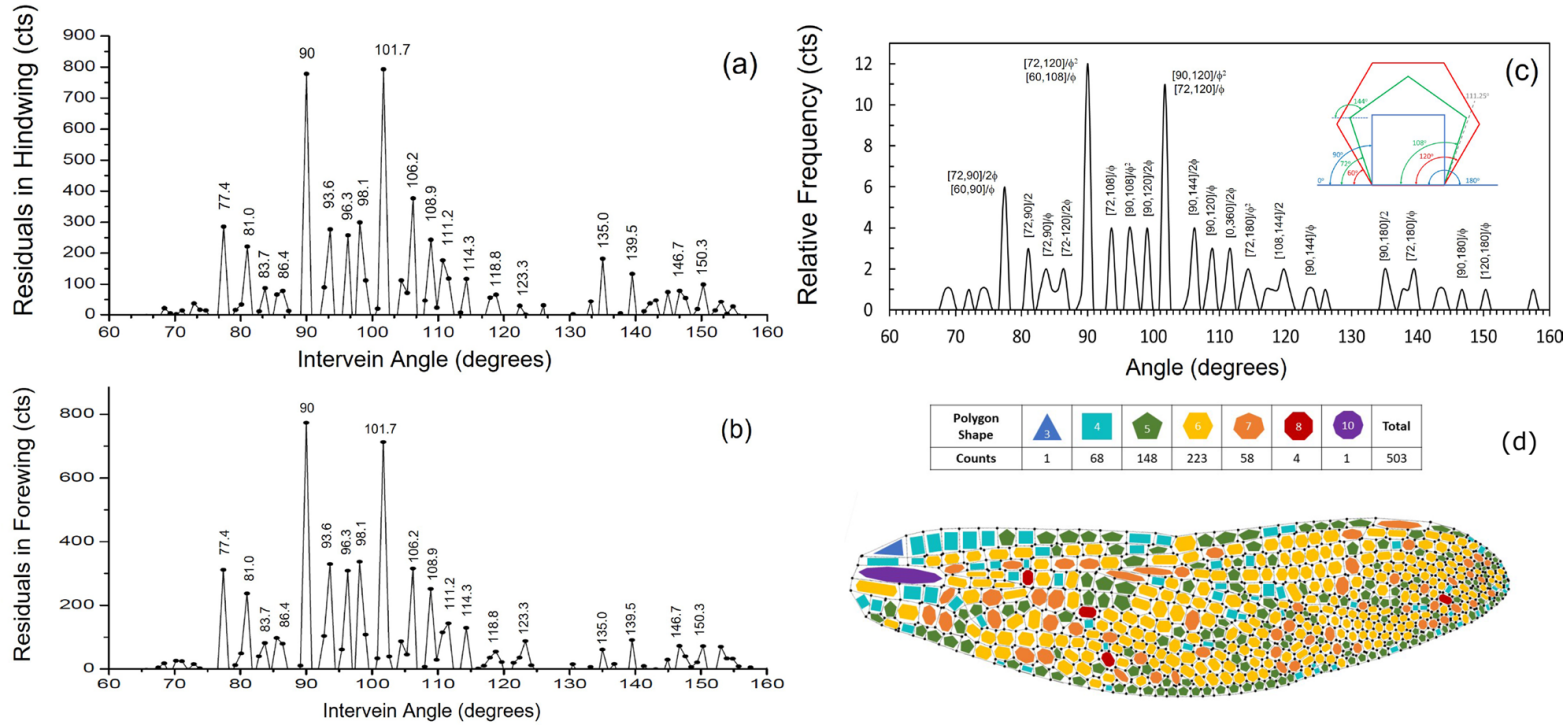
Residuals from the Gaussian-peak fitting for the hindwing (**a**) and forewing (**b**). The angle positions of the strong residuals are indicated in the figures, and they appear to be identical for both forewing and hindwing. (**c**) Relative occurrence frequencies of the predicted preferred angles given by golden-rule partitions of the intervals between regular angle pairs (Eq. 1) in perfectly shaped quadrilateral, pentagon, and hexagon venation cells (see inset). (**d**) Polygon shapes in the venation pattern of a dragonfly wing. It shows that hexagons, pentagons, and quadrilaterals are the most popular shapes in the pattern.

The fitting residuals or outliers well above the level that can be interpreted by statistical random errors are shown in Fig. 4a,b for the hindwings and forewings, respectively. Several observations can be made for these strong residuals. Firstly, both the forewing and the hindwing patterns show an identical set of intervein angles for their residuals, despite the geometric overall shapes being very different (Fig. 2 inset) for the forewing and the hindwing. This is a clear indication that these outliers are not due to random statistical fluctuations. Secondly, almost all strong residuals are located between $75^{\circ }$ and $120^{\circ }$, with the two strongest ones at $90^{\circ }$ and $101^{\circ }$.

## Golden-ratio partition model

We have developed an empirical Golden-Ratio Partition model to interpret these preferred intervein angles in the dragonfly wings venation patterns. Our model is inspired by a method proposed by Takuya Okabe for the interpretation of phyllotaxis patterns in leaf growth in a variety of plants^28^. In Okabe’s work, a new adaptive mechanism was proposed based on the principle that optimization of the divergent angles between plant leave stems leads to minimization of the energy cost of the phyllotaxis transition. This model can explain not only the presence of the golden angle but also the occurrence of other angles such as the Fibonacci number ratios observed in nature.

In our dragonfly wing case, the purpose of the venation pattern optimization is to use the least amount of support veins that support the very-thin membrane to minimize the weight of the wing, while still providing the biomechanical and aerodynamic functions that are required for a dragonfly to fly. This leads to the following considerations in our model.

First, for a given area in unconstrained space, the minimum line boundary length is a circle. However, it is impossible to pack circles in space efficiently. Thus, a circular shape can be approximated by polygons such as hexagons for honeycombs in beehives. The formation of the venation pattern on a dragonfly wing is very different, as their formation is constrained by the overall wing boundary that is shaped to be aerodynamically efficient. Therefore, the venation veins must be developed within this constraint, which means it would be impossible to have regular polygon patterns as in the case of the honeycombs.

Second, we hypothesize that it is this boundary constraint that forces the formation of irregular-shaped or distorted polygons in the venation vein patterns in the dragonfly wings. The distorted polygons follow the golden-ratio rule ($\phi$ = 1.618) and low-order Fibonacci number series as rational approximates (1/2, 2/3, 3/5, …, which approaches its limit $1/\phi = 0.618$) to partition the angle intervals defined by $[\alpha _i, \alpha _j]$, where $[\alpha _i, \alpha _j]$ are two regular angles either within the same polygon group (e.g. 72$^{\circ }$ and 108$^{\circ }$ for pentagons) or from two different polygon groups (e.g. 72$^{\circ }$ and 120$^{\circ }$ between a pentagon and a hexagon). These regular angles are illustrated in Fig. 4c inset.

Finally, a closer look at the venation patterns in dragonfly wings indicates that the most popular shapes by far are hexagons and pentagons, followed by quadrilaterals, as shown in Fig. 4d. We argue that heptagons and higher-order polygons would mostly contribute to the broad peaks centered at 111.25$^{\circ }$ and 137.5$^{\circ }$ as they will contribute many angular intervals that eventually give rise to the broad distribution of the intervein angles discussed in the previous section. Therefore, in our analysis of the outliers, we will only consider the regular angles associated with the hexagon, pentagon, and quadrilateral shapes.

With the model outlined above, we can now estimate a set of preferred intervein angles $\alpha$ in the venation pattern from the regular polygon angle intervals $[\alpha _i, \alpha _j]$ and the partition ratio *p*/*q*, which equals the golden ratio $1/\phi$ and the low-order Fibonacci rational approximates 1/2, 2/3, 3/5:

1 $$\begin{aligned} \alpha =[\alpha _i, \alpha _j] p/q = \alpha _i + (\alpha _j - \alpha _i) p/q , \end{aligned}$$

where $p/q = 1/2, 2/3, 3/5$, …, $1/\phi$, and $[\alpha _i, \alpha _j]$ equals to any two regular angles in perfect rectangles (0$^{\circ }$, 90$^{\circ }$, 180$^{\circ }$), pentagons (72$^{\circ }$, 108$^{\circ }$, 144$^{\circ }$), and hexagons (60$^{\circ }$, 120$^{\circ }$). Using this method, in Fig. 4c we plot the calculated preferred angle locations from Eq. 1, where each occurrence is labeled by a specific $[\alpha _i, \alpha _j] p/q$ that produced that angle. In the calculation, we include two primary second-level partition ratios with the lowest orders ($1/\phi ^2, 1/2\phi$). Since our angle distribution histogram is sampled with an angular interval of $0.9^{\circ }$, we use the same set of angle values to position our calculated preferred angles. In addition, if an angle $\alpha$ calculated by Eq. 1 is within $1.4^{\circ }$ of the location of a residual peak in the measured histogram, then the angle $\alpha$ is counted in the angle interval of that residual peak. This is because the histogram angular interval $0.9^{\circ }$ and the statistical uncertainty $\pm 1^{\circ }$ in the least-squares regression fits add quadratically as the total statistical error on the angular positions.

As also shown in Fig. 4c, our approach not only predicts the angular positions of all observed strong residuals in the intervein angle histograms, but also provides relative frequencies of their occurrences thus their peak heights. This is because the preferred angles defined by Eq. 1 for different angle pairs may overlap for certain angles, making these angles more likely to occur than others with greater probability. As an example, $\alpha \{[90, 120]/\phi ^2\} = 90 +(120-90)/1.618^2 = 101.5^{\circ }$, and $\alpha \{[72, 120]/\phi \} = 72 +(120-72)/1.618 = 101.7^{\circ }$, both contribute to the residual peak at $101.7^{\circ }$, making it twice likely to occur compared to a residual to which only one $\alpha \{[\alpha _i, \alpha _j] p/q\}$ contributes. Summing up all possible occurrences, the peak height for each $\alpha$ is shown in Fig. 4c. As one can see, Fig. 4c agrees reasonably well with the residuals plots in Fig. 4a,b, in both the locations and the heights of the residual peaks, suggesting that our model is a good way to describe the key features in the venation patterns of dragonfly wings, and how these venation patterns should be formed in constrained space.

## Conclusion

In summary, the naturally optimized venation structure in dragonfly wings was explored by studying the angular distribution of vein struts. We found that the golden rule plays a prominent role in the formation of the venation patterns in dragonfly wings. First, the most pronounced angle combination was directly related to the golden angle, which is known to play a critical role in structural optimization in nature. The venation intersections that utilize the golden angle tend to concentrate near the trailing edges and wing tips. In addition, we found that there exist a set of preferred intervein angles in the venation patterns that are the results of golden-rule partitions of regular-polygon angles under the constraint of the confined space. The venation patterns that exhibit these preferred angles appear in broad areas on the wings, including near the edge regions of the wings where primary veins are formed and transition into secondary vein structures. These observations provide further insight into the golden-rule optimization process that exists in nature, which has been widely shown to optimize structural integrity and biomechanical function, and has now also been demonstrated for the dragonfly wing.

## Data Availability

The datasets used and/or analyzed during the current study are available from the corresponding author upon reasonable request.
